# Postoperative Antibiotic Escalation After Major Free-Flap Reconstruction Requiring ICU Admission: Associations with Day-1 Procalcitonin, Shock, and Microbiological Positivity

**DOI:** 10.3390/life16020204

**Published:** 2026-01-26

**Authors:** Wei-Hung Chang, Kuang-Hua Cheng, Ting-Yu Hu, Hui-Fang Hsieh, Kuan-Pen Yu

**Affiliations:** 1 Department of Critical Care Medicine, MacKay Memorial Hospital, No. 92, Sec. 2, Zhongshan North Road, Taipei 10449, Taiwan; peacejaycool@gmail.com (W.-H.C.); jeff01@mmh.org.tw (K.-H.C.);; 2 Department of Nursing, MacKay Memorial Hospital, Taipei 104, Taiwan

**Keywords:** free-flap reconstruction, antibiotic escalation, procalcitonin, postoperative shock, surgical ICU, antimicrobial stewardship, microbiological positivity

## Abstract

Major reconstructive free-flap surgery often requires ICU admission, yet early signals associated with postoperative antibiotic escalation remain poorly characterized. We conducted a single-center retrospective cohort study of 119 consecutive postoperative ICU admissions after major free-flap reconstruction. Exposures were postoperative day-1 procalcitonin (PCT) and documented postoperative shock; the primary endpoint was clinician-initiated antibiotic escalation (“upgrade”), and secondary endpoints were documented microbiological positivity and ICU mechanical ventilation duration. Escalation occurred in 85/119 admissions (71.4%). Day-1 PCT was higher with escalation (median 0.25 vs. 0.135 ng/mL; *p* = 0.033), and shock was more frequent (59/85 [69.4%] vs. 13/34 [38.2%]; *p* = 0.003). Escalation was associated with longer ventilation (median 3515 vs. 2170 min; *p* < 0.001) and higher rates of any positive culture (54/85 [63.5%] vs. 8/34 [23.5%]; *p* < 0.001). In multivariable logistic regression adjusting for operative time and intraoperative IV volume, shock remained independently associated with escalation (adjusted OR 3.52, 95% CI 1.48–8.36; *p* = 0.004), whereas log-transformed PCT was not (*p* = 0.224). PCT showed modest apparent discrimination for escalation (AUC 0.63), improving to 0.71 when combined with shock. These findings should be interpreted as observational associations with escalation behavior, supporting prospective evaluation of physiology-plus-biomarker stewardship approaches.

## 1. Introduction

Major reconstructive surgery using microvascular free-tissue transfer (free flaps) is a cornerstone for restoring complex defects, particularly after oncologic resection in the head and neck region. Postoperative infectious and wound-related complications remain frequent and clinically consequential; in a post hoc analysis of 697 patients included in a systematic review of clean-contaminated head and neck free-flap reconstruction, donor-site surgical site infection occurred in 10.6% and distant infections (pneumonia or urinary tract infection) occurred in 17.9% [1]. Preventing and promptly addressing these complications is central to postoperative care because they can increase morbidity and prolong recovery in major head and neck oncologic surgery [2].

Antibiotic prophylaxis is a core component of infection prevention in this setting, yet optimal regimens remain debated. In clean-contaminated head and neck surgery, surgical site infection rates without antibiotic prophylaxis have been reported to range from 24% to 87%, and microvascular reconstruction appears particularly prone to infection [3]. Systematic reviews focusing on major head and neck cancer surgery with flap reconstruction highlight substantial heterogeneity in antibiotic selection and duration, and the evidence base has not fully resolved the balance between adequate coverage and antimicrobial stewardship [4].

General surgical prophylaxis guidelines recommend selecting agents that cover expected flora and limiting prophylaxis duration to reduce adverse effects and antimicrobial resistance pressure [5]. Complementary prevention frameworks emphasize that antibiotic prophylaxis is only one element within broader perioperative bundles designed to reduce surgical site infections [6].

Despite prophylaxis, early postoperative physiologic derangements and nonspecific inflammatory responses often create diagnostic uncertainty, especially in patients requiring ICU admission after complex reconstruction. In this setting, concern for postoperative pneumonia and other nosocomial infections is heightened, and ventilator-associated respiratory infection pathways may shape clinicians’ thresholds for broadening antimicrobial therapy [7]. At the same time, ICU stewardship programs emphasize timely diagnostic sampling, structured reassessment, and de-escalation to avoid unnecessary broad-spectrum exposure [8].

Hemodynamic instability is a particularly high-impact trigger for empiric antibiotic escalation. Consensus frameworks define circulatory shock as a state of inadequate tissue perfusion that can arise from diverse etiologies, underscoring that postoperative “shock” is not synonymous with infection [9]. Consequently, when clinician-initiated antibiotic escalation is used as an endpoint in observational work, associations with severity markers may reflect both true infection risk and confounding by indication.

Procalcitonin (PCT) has been proposed as an adjunct biomarker to support antibiotic decision-making in critical illness. In ICU trials, PCT-guided algorithms reduced antibiotic exposure compared with standard care [10]. Additional randomized studies have supported PCT-guided shortening of antibiotic courses in critically ill patients [11]. These approaches typically rely on serial measurements and predefined reassessment rules rather than a single early value [12]. In major head and neck surgery, perioperative studies suggest that postoperative PCT kinetics can correlate with complications such as pharyngocutaneous fistula, but interpretation in the immediate postoperative period remains complex [13]. A systematic review and meta-analysis further supports an association between postoperative PCT and fistula-related outcomes, reinforcing the need to interpret PCT in clinical context rather than in isolation [14]. Large head and neck free-flap cohorts have also documented substantial postoperative infection burdens and variability in prophylaxis practices, illustrating ongoing opportunities to align clinical risk signals with stewardship principles [15].

The incremental contribution of this study lies in characterizing real-world antibiotic escalation behavior in a high-risk surgical ICU free-flap population, rather than proposing a new biomarker-based decision rule.

Therefore, we conducted a single-center retrospective cohort study of consecutive ICU admissions after major free-flap reconstruction. The primary objective was to evaluate the associations of postoperative day-1 procalcitonin (PCT) and documented postoperative shock with postoperative antibiotic escalation (“upgrade”). Secondary objectives were to examine the associations between antibiotic escalation and microbiological positivity as well as ICU mechanical ventilation duration.

In this cohort, antibiotic escalation was frequent and was more strongly associated with early postoperative shock than with a single day-1 PCT value, while escalation also coincided with higher microbiological positivity and longer mechanical ventilation duration.

## 2. Materials and Methods

### 2.1. Study Design, Setting, and Ethical Approval

We conducted a single-center retrospective cohort study of postoperative patients admitted to the intensive care unit (ICU) after major reconstructive free-flap surgery at MacKay Memorial Hospital (Taipei, Taiwan). The study protocol was reviewed and approved by the Institutional Review Board of MacKay Memorial Hospital (protocol code 21MMHIS430e). Because this was a retrospective analysis of routinely collected clinical data, informed consent was waived by the IRB. The manuscript was prepared in accordance with reporting recommendations for observational studies.

### 2.2. Study Population and Data Source

The analytic cohort comprised 119 consecutive ICU admissions following major reconstructive (free-flap) surgery captured in the study dataset. Each ICU admission was treated as one analytic episode. If a patient underwent more than one eligible surgery resulting in separate ICU admissions, each admission was analyzed as a separate episode because perioperative exposures and postoperative course differed by admission. Data were abstracted from routinely documented perioperative and ICU records and entered into a structured dataset containing operative durations, intraoperative fluid balance metrics, postoperative laboratory values, ICU respiratory support duration, and postoperative clinical events. Among 119 ICU admissions, there were 118 unique patients; only one patient contributed two admissions. Therefore, within-patient clustering was minimal.

### 2.3. Variables and Measurements

Perioperative variables included total operative time (minutes), intraoperative intravenous fluid volume, intraoperative urine output, estimated intraoperative blood loss, and intraoperative diuretic exposure (recorded as the number of furosemide/Lasix ampoules administered). Postoperative variables included ICU mechanical ventilation duration (minutes) and postoperative day-1 procalcitonin (PCT; ng/mL) measured as part of routine postoperative ICU care.

Postoperative clinical events (e.g., shock, vasopressor use, antibiotic escalation, and culture positivity) were recorded as binary indicators within the dataset. For these event variables, a positive label (e.g., “shock”, “inotropics”, “upgrade”, “yes”) indicated the event occurred; absence of a positive label was treated as no recorded event. For key clinician-driven events (shock and antibiotic escalation), variables were derived from routine clinical documentation and medication records as captured in the structured dataset.

### 2.4. Exposure and Outcome Definitions

The primary exposure of interest was postoperative day-1 PCT. Because PCT values were right-skewed, PCT was analyzed descriptively using non-parametric summaries and was log-transformed (natural logarithm) for regression modeling to reduce the influence of extreme values.

Documented postoperative shock was defined operationally based on routine ICU documentation as a binary event during the early postoperative period (postoperative day 1). Vasopressor use was captured as a separate binary variable in the dataset. Granular physiologic thresholds (e.g., mean arterial pressure cutoffs, lactate) and shock etiology (septic vs. hemorrhagic vs. vasoplegic) were not uniformly available and therefore could not be adjudicated.

For admissions labeled as shock, the dataset additionally recorded the time from postoperative time zero to the first documented shock event; this timing variable was used descriptively only. Postoperative time zero was defined in the dataset as the end of surgery.

The primary outcome was postoperative antibiotic escalation, operationally defined as any broadening or intensification of systemic antimicrobial coverage recorded during the postoperative ICU course, captured in the dataset as an “upgrade”. Because the dataset did not include baseline prophylactic/empiric regimens, antibiotic classes, dosing, or exact timing of escalation, this endpoint should be interpreted as a clinician-driven escalation event rather than a microbiologically confirmed outcome.

Secondary outcomes included microbiological positivity, defined as documented growth (‘yes’) in any of the recorded culture categories (sputum, wound, and/or blood). Culture variables were available as indicators of documented growth; negative culture results and cultures not obtained could not be distinguished and were therefore treated as ‘no documented growth’ for analysis. Because culture acquisition is clinician-driven and more likely in clinically deteriorating patients, associations involving microbiological positivity should be interpreted cautiously due to potential verification and sampling bias.

### 2.5. Statistical Analysis

Continuous variables were summarized as median (interquartile range, IQR) and compared between groups using the Mann–Whitney U test. Categorical variables were summarized as counts (percentages) and compared using the chi-square test or Fisher’s exact test when appropriate.

To evaluate independent associations with antibiotic escalation, we fitted multivariable logistic regression models with antibiotic escalation (yes/no) as the dependent variable. The prespecified covariates included postoperative shock (binary), log-transformed day-1 PCT, and key intraoperative covariates available in the dataset that may confound escalation decisions and postoperative trajectory (total operative time and intraoperative intravenous fluid volume). Effect sizes were reported as odds ratios (ORs) with 95% confidence intervals (CIs).

Apparent discrimination for antibiotic escalation was assessed using receiver operating characteristic (ROC) analysis, reporting the area under the ROC curve (AUC). AUCs were calculated for PCT alone and for combined models (e.g., shock plus PCT) using model-predicted probabilities; these ROC analyses were exploratory and were not accompanied by internal validation or calibration assessment.

### 2.6. Data and Code Availability

The dataset contains patient-level clinical information and is not publicly available due to privacy and institutional restrictions. De-identified data and analytic code may be made available from the corresponding author upon reasonable request, subject to institutional approvals and applicable regulations.

## 3. Results

### 3.1. Study Cohort and Frequency of Antibiotic Escalation

**Figure 1:**
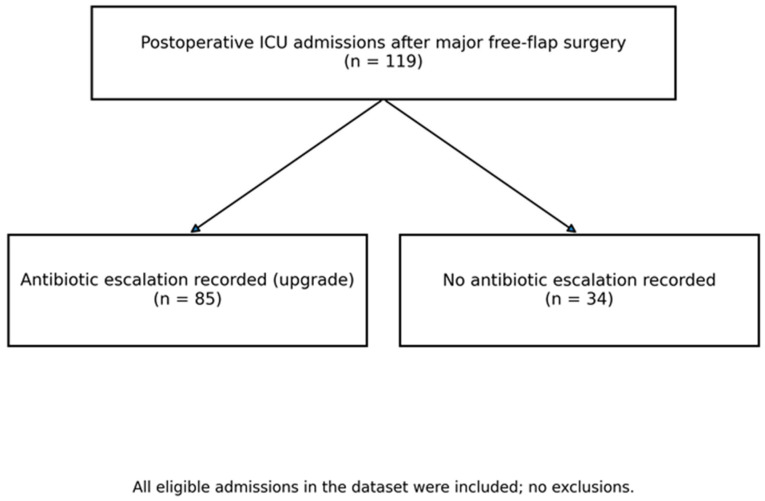
Flow diagram of cohort inclusion. A total of 119 consecutive postoperative ICU admissions after major free-flap reconstruction were included (118 unique patients; one patient contributed two admissions). No admissions were excluded. Postoperative day-1 procalcitonin was available for all included admissions (119/119).

The cohort comprised 119 consecutive postoperative ICU admissions after major free-flap reconstruction. Antibiotic escalation (recorded as ‘upgrade’) occurred in 85/119 admissions (71.4%). Figure 1 summarizes cohort inclusion and group allocation. Perioperative and early postoperative characteristics by antibiotic escalation status are summarized in Table 1.

### 3.2. Perioperative Factors and Early Postoperative Signals

**Figure 2:**
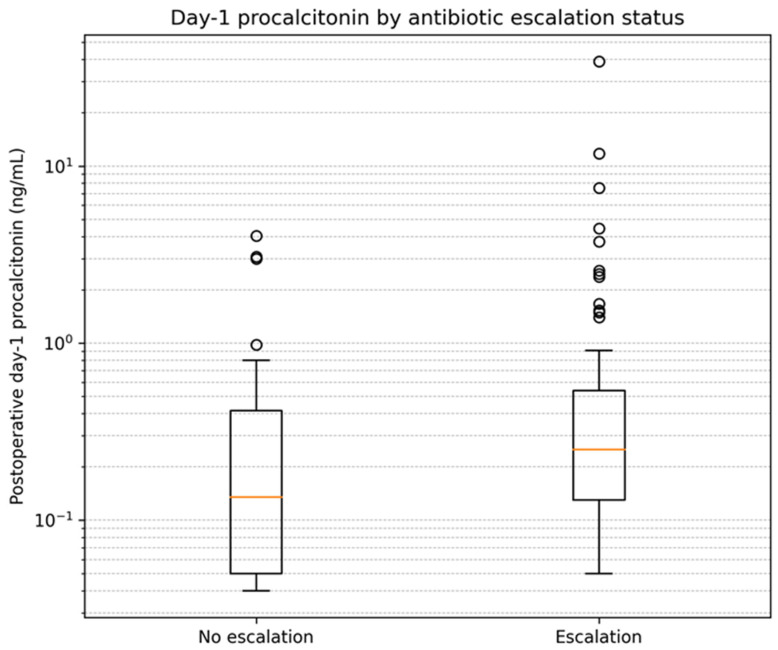
Postoperative day-1 procalcitonin by antibiotic escalation status. The y-axis is logarithmic.

Compared with patients without escalation, those with antibiotic escalation had longer operative times (805 (705–914) vs. 766 (666–822); *p* = 0.028) and received higher intraoperative IV fluid volumes (5350 (4450–6650) vs. 4725 (3525–5650); *p* = 0.014). Postoperative day-1 procalcitonin was higher in the escalation group (0.250 (0.130–0.540) vs. 0.135 (0.050–0.415) ng/mL; *p* = 0.033). Documented postoperative shock and vasopressor use were also more frequent in the escalation group (shock: 59 (69.4%) vs. 13 (38.2%); *p* = 0.003; vasopressors: 22 (25.9%) vs. 1 (2.9%); *p* = 0.009). Among admissions with documented postoperative shock, the median time from postoperative time zero to the first recorded shock event was 452.5 min (interquartile range, 257.5–662.5). The distribution of postoperative day-1 procalcitonin by antibiotic escalation status is shown in Figure 2.

### 3.3. Microbiological Findings and ICU Outcomes

Microbiological positivity was more common among patients who underwent antibiotic escalation. Any documented positive culture (sputum, wound, or blood) occurred in 54 (63.5%) compared with 8 (23.5%) (*p* < 0.001). Sputum culture positivity (39 (45.9%) vs. 7 (20.6%); *p* = 0.019) and wound culture positivity (30 (35.3%) vs. 1 (2.9%); *p* < 0.001) were both higher in the escalation group, whereas blood culture positivity was uncommon and did not differ significantly between groups (6 (7.1%) vs. 0 (0.0%); *p* = 0.181). Patients with antibiotic escalation required longer mechanical ventilation in the ICU (3515 (3305–5610) vs. 2170 (1972–2557); *p* < 0.001). Microbiological results and ICU outcomes are summarized in Table 2.

### 3.4. Multivariable Association and Discrimination

**Figure 3:**
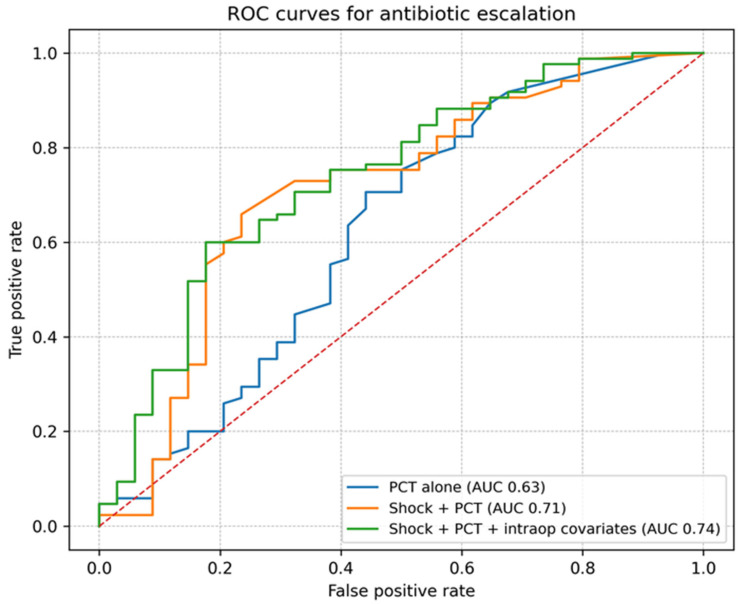
ROC curves showing apparent discrimination for antibiotic escalation. Curves are shown for postoperative day-1 procalcitonin alone, shock plus procalcitonin, and the multivariable model including intraoperative covariates. AUCs represent apparent performance in this retrospective cohort.

In multivariable logistic regression adjusted for operative time and intraoperative IV volume, documented postoperative shock remained independently associated with antibiotic escalation (adjusted OR 3.52, 95% CI 1.48–8.36; *p* = 0.004), whereas day-1 procalcitonin (log-transformed) was not independently associated with escalation (adjusted OR 1.24, 95% CI 0.88–1.76; *p* = 0.224). Apparent discrimination for escalation was modest using day-1 PCT alone (AUC 0.63) and improved when combined with postoperative shock (AUC 0.71); the model additionally including intraoperative covariates yielded an apparent AUC of 0.74 (Figure 3). Multivariable logistic regression results are presented in Table 3.

## 4. Discussion

In this retrospective ICU cohort of postoperative major free-flap reconstruction, antibiotic escalation was common and clustered with early indicators of clinical instability. Patients with escalation had higher postoperative day-1 procalcitonin (PCT), substantially more documented postoperative shock and vasopressor exposure, more frequent microbiological positivity, and markedly longer duration of mechanical ventilation. These findings support the premise that, in high-risk reconstructive surgical ICU patients, antibiotic escalation is often driven by early physiologic deterioration and concern for infectious complications that may jeopardize both systemic recovery and reconstructive outcomes [16]. Importantly, antibiotic escalation in this study represents a clinician-driven decision under postoperative diagnostic uncertainty rather than a microbiologically confirmed outcome. Accordingly, the following findings should be interpreted as associations with escalation behavior, not as predictors of infection or clinical outcomes.

### 4.1. Antibiotic Escalation as a Pragmatic, Clinically Meaningful Endpoint

Antibiotic escalation in the immediate postoperative period is not solely a microbiology-driven action; it represents a real-world decision under uncertainty, balancing the consequences of missed infection against antimicrobial overuse. In free-flap reconstruction, where wound complications can carry high morbidity and threaten reconstructive success, clinicians may have a lower threshold to broaden coverage when early warning signs appear [17]. From a systems perspective, escalation is therefore a pragmatic surrogate for high-risk postoperative trajectories and stewardship-relevant antibiotic exposure rather than a direct synonym for confirmed infection.

### 4.2. Stewardship Tension in Surgical ICU Patients

ICU antibiotic use is consistently high, and unnecessary broad-spectrum exposure increases the risk of adverse drug events, Clostridioides difficile infection, selection of resistant organisms, and downstream colonization pressure [18]. Contemporary stewardship frameworks emphasize early diagnostic sampling, timely initiation when infection is suspected, and structured reassessment with de-escalation when infection is not supported [19]. Our data illustrate the practical tension in postoperative free-flap ICU care: escalation was frequent, yet the determinants of escalation reflected severity signals (especially shock) more than a single biomarker, suggesting that decision-making remains dominated by bedside physiology and perceived risk rather than biomarker thresholds alone.

Practical implication: In high-risk postoperative free-flap ICU care, a stewardship-aligned workflow may emphasize (i) early standardized microbiological sampling at the time of clinical deterioration, (ii) explicit reassessment at 48–72 h to confirm ongoing infectious justification, and (iii) active de-escalation when cultures and clinical trajectory do not support infection. In this context, PCT should be interpreted as an adjunct signal that may inform reassessment rather than a stand-alone trigger for escalation.

### 4.3. Interpreting Pct in the Postoperative Setting

PCT has biologic plausibility as a marker of bacterial infection and sepsis, but postoperative inflammatory responses, tissue trauma, and perioperative physiologic stress can affect its kinetics, complicating single-time-point interpretation [20]. Meta-analytic evidence supports PCT’s diagnostic utility in critical illness, yet performance varies by population, infection source, and timing of measurement [21]. In our cohort, day-1 PCT was statistically higher in patients who underwent escalation; however, PCT did not remain independently associated with escalation after adjustment, indicating that in this setting PCT may provide incremental information but is not the primary driver once overt instability is present.

### 4.4. How Our Findings Fit with Pct-Guided Antibiotic Strategies

Randomized and meta-analytic data suggest that PCT-guided strategies can reduce antibiotic exposure in selected respiratory and ICU populations without compromising safety, particularly when used to support reassessment and discontinuation decisions rather than as a stand-alone diagnostic trigger [22]. Many guideline and consensus statements accordingly position PCT as an adjunct to clinical judgment—useful for stewardship when interpreted in context and when paired with active reassessment [23]. Our results align with that framing: PCT alone showed only modest discrimination for escalation, while combining PCT with shock improved discrimination, implying that a hybrid “physiology + biomarker” approach may be more operationally useful than either component alone.

### 4.5. Why the Absolute Pct Signal May Be Modest in Free-Flap ICU Care

The absolute difference in day-1 PCT between groups occurred within a range that may overlap with postoperative inflammatory responses, potentially blunting actionable separation. This is consistent with perioperative head-and-neck surgical series suggesting that postoperative PCT is influenced by surgical burden and may be more informative when interpreted dynamically (serial change) rather than as a single value [24]. Moreover, commonly cited PCT thresholds for bacterial infection may not translate directly to complex reconstructive surgery, where tissue injury and transfusion/physiologic stress can confound interpretation [25]. These factors likely contribute to the observation that PCT added incremental, but limited, standalone discriminatory value for escalation in our cohort.

### 4.6. Shock as the Dominant Escalation Trigger and the Risk of Noninfectious Confounding

Postoperative shock was strongly associated with escalation and remained independently associated in multivariable analysis. This is clinically intuitive: hemodynamic instability prompts urgent rule-out and empiric treatment for infection. However, postoperative shock in prolonged reconstructive surgery can arise from multiple noninfectious mechanisms, including bleeding, anesthetic-related vasodilation, vasoplegia, hypovolemia, and cardiopulmonary complications [26]. Given the high stakes of delayed therapy in true septic shock, clinicians may appropriately adopt an “act first” posture when shock is present [27]. Our findings therefore likely reflect a combination of appropriate early empiricism in high-risk patients and unavoidable confounding by indication, where the physiologic severity that triggers escalation is not always infectious in origin. This confounding-by-indication structure also implies that shock and other severity signals are simultaneously determinants of escalation and correlates of worse trajectories, limiting causal interpretation in an observational design.

### 4.7. Microbiological Positivity: Supportive Signal with Important Caveats

Microbiological positivity was substantially more frequent among patients with escalation, particularly for sputum and wound cultures. This supports that escalation often coincided with objective microbiological findings, but interpretation requires caution. Postoperative wound and respiratory cultures can reflect colonization or sampling bias (cultures obtained more frequently in clinically deteriorating patients), and a “no growth” label in routine data may represent either a true negative result or a culture not obtained [28]. In ventilated postoperative ICU patients, guideline-based diagnostic pathways for hospital-acquired and ventilator-associated pneumonia emphasize careful clinical correlation because respiratory culture positivity alone does not establish infection [29]. These caveats reinforce that microbiological signals should be integrated with clinical course rather than used in isolation to justify continued broad coverage. Consequently, the observed association between antibiotic escalation and microbiological positivity should not be interpreted as evidence that escalation was microbiologically justified. Rather, it likely reflects differential testing practices and verification/sampling bias, because clinically deteriorating patients are more likely to have cultures obtained.

### 4.8. Link to Respiratory Support Burden and Perioperative Complications

Patients with escalation had markedly longer mechanical ventilation duration, consistent with a clinical phenotype of greater postoperative physiologic derangement and/or pulmonary complications. Prolonged ventilation is a known risk factor for ventilator-associated respiratory infections and is associated with worse ICU outcomes, creating a feedback loop in which instability, ventilator dependence, and infection risk reinforce each other [30]. In free-flap patients, prolonged sedation and airway protection strategies can further increase aspiration risk and complicate early mobilization, potentially contributing to infectious trajectories that prompt escalation [31]. While causality cannot be inferred here, the association underscores the importance of integrated postoperative care pathways addressing hemodynamics, respiratory management, and infection surveillance together rather than as separate silos.

### 4.9. Implications for Risk Stratification and Model Development

From an exploratory standpoint, our ROC analyses describe apparent discrimination for antibiotic escalation, with limited separation by PCT alone and modest improvement when combined with shock and basic intraoperative covariates. This supports a practical direction: rather than searching for a single “silver bullet” biomarker, a parsimonious, clinically grounded multivariable model may better reflect the real decision drivers behind escalation. Future model development should follow best practices for prediction research, including transparent reporting, avoidance of overfitting, and external validation before clinical adoption [32]. In addition, discrimination metrics should be interpreted with appropriate caution; moderate AUC improvements may not translate into safe decision thresholds without calibration assessment and prospective evaluation [33]. These AUC estimates reflect apparent performance in a single retrospective cohort and should not be interpreted as a validated predictive tool in the absence of internal validation and calibration assessment.

### 4.10. Limitations and Future Directions

This study has several limitations. First, its retrospective single-center design and the use of operational, documentation-based variables (e.g., “shock,” “upgrade”) introduce misclassification risk and confounding by indication; escalation may be influenced by clinician preference, service-level protocols, or perceived flap risk rather than infection alone [34]. Second, culture data were captured as positivity indicators and could not distinguish “not obtained” from “obtained and negative,” limiting inference regarding diagnostic yield. Third, PCT was analyzed as day-1 measurement; serial kinetics, time from surgery to sampling, and concurrent inflammatory markers were not incorporated and may provide stronger decision support. In addition, severity-of-illness scores (e.g., SOFA, APACHE II), conventional inflammatory markers (e.g., white blood cell count, C-reactive protein), and renal/hepatic function variables were not available in the structured dataset and could not be evaluated as confounders or comparators. These data limitations may contribute to residual confounding and preclude direct benchmarking of PCT against widely used markers.

Future work should prospectively test an integrated escalation/de-escalation pathway combining bedside physiology (e.g., shock/vasopressor requirement), structured microbiological sampling, and serial biomarker trends, with predefined reassessment windows to support early narrowing when infection is not supported [35]. Prospectively collected serial PCT trajectories may better distinguish postoperative inflammatory responses from evolving bacterial infection than a single day-1 value, and could be integrated into predefined reassessment windows to support de-escalation. Such a pathway would directly target the operational problem highlighted here: frequent escalation under uncertainty, with the goal of preserving patient safety while improving stewardship in this high-risk reconstructive surgical ICU population.

This study was not designed to compare procalcitonin against conventional markers such as white blood cell count, C-reactive protein, or severity-of-illness scores (SOFA/APACHE II); future prospective studies should directly compare these measures and assess whether serial PCT trajectories provide incremental stewardship value beyond established severity and inflammatory indicators.

## 5. Conclusions

In this high-risk surgical ICU free-flap cohort, clinician-initiated antibiotic escalation was frequent and was most strongly associated with early postoperative shock. Postoperative day-1 procalcitonin showed modest apparent discrimination and should be interpreted as an adjunct signal in an observational context rather than as a stand-alone predictor. These results are hypothesis-generating and provide groundwork for prospective stewardship algorithms incorporating serial biomarkers, standardized diagnostic sampling, and predefined reassessment to support timely de-escalation.

## Figures and Tables

**Table 1:** Perioperative and early postoperative characteristics by antibiotic escalation status.

Variable	Overall (n = 119)	No Escalation (n = 34)	Escalation (n = 85)	*p* Value
Total operative time, min	782 (699–892)	766 (666–822)	805 (705–914)	0.028
Intraoperative IV volume, mL	5170 (4100–6500)	4725 (3525–5650)	5350 (4450–6650)	0.014
Intraoperative urine output, mL	1500 (1055–2090)	1280 (985–2000)	1580 (1100–2100)	0.412
Estimated blood loss, mL	350 (200–550)	250 (200–488)	400 (250–550)	0.082
Postoperative day-1 procalcitonin, ng/mL	0.220 (0.100–0.530)	0.135 (0.050–0.415)	0.250 (0.130–0.540)	0.033
Postoperative shock, n (%)	72 (60.5%)	13 (38.2%)	59 (69.4%)	0.003
Postoperative vasopressor use, n (%)	23 (19.3%)	1 (2.9%)	22 (25.9%)	0.009

Data are presented as median (IQR) or n (%). *p* values are from Mann–Whitney U tests for continuous variables and chi-square/Fisher’s exact tests for categorical variables. Abbreviations: IQR, interquartile range; IV, intravenous.

**Table 2:** Microbiological results and ICU outcomes by antibiotic escalation status.

Variable	Overall (n = 119)	No Escalation (n = 34)	Escalation (n = 85)	*p* Value
Any positive culture (sputum, wound, or blood), n (%)	62 (52.1%)	8 (23.5%)	54 (63.5%)	<0.001
Sputum culture positive, n (%)	46 (38.7%)	7 (20.6%)	39 (45.9%)	0.019
Wound culture positive, n (%)	31 (26.1%)	1 (2.9%)	30 (35.3%)	<0.001
Blood culture positive, n (%)	6 (5.0%)	0 (0.0%)	6 (7.1%)	0.181
ICU mechanical ventilation duration, min	3405 (2125–4808)	2170 (1972–2557)	3515 (3305–5610)	<0.001

Data are presented as median (IQR) or n (%). *p* values are from Mann–Whitney U tests for continuous variables and chi-square/Fisher’s exact tests for categorical variables. Abbreviations: ICU, intensive care unit; IQR, interquartile range.

**Table 3:** Multivariable logistic regression for antibiotic escalation.

Predictor	Adjusted OR	95% CI	*p* Value
Postoperative shock (yes vs. no)	3.52	1.48–8.36	0.004
Day-1 procalcitonin (log-transformed)	1.24	0.88–1.76	0.224
Total operative time (per hour)	1.14	0.89–1.45	0.296
Intraoperative IV volume (per 1 L)	1.16	0.82–1.64	0.410

Adjusted odds ratios (ORs) are from a logistic regression model including postoperative shock, log-transformed day-1 procalcitonin, total operative time (per hour), and intraoperative IV volume (per 1 L). Abbreviations: OR, odds ratio; CI, confidence interval; IV, intravenous.

## Data Availability

The data that support the findings of this study are available from the corresponding author upon reasonable request. The data are not publicly available due to institutional and ethical restrictions related to patient privacy.

## Abbreviations

ICU	intensive care unit
PCT	procalcitonin
ROC	receiver operating characteristic
AUC	area under the curve
OR	odds ratio
IQR	interquartile range
